# Sea-Sickness and Its Prevention

**Published:** 1883-10

**Authors:** Henry J. Bennet

**Affiliations:** Formerly Obstetric Physician to the Royal Free Hospital


SEA-SICKNESS AND ITS PREVENTION. BY HENRY J. BENNET, M.D., Formerly Obstetric Physician to the Royal Free Hospital.

In his interesting letter on sea-sickness which appeared in the *Journal* of July 7th, Mr. Kendall mentions various modes of treating sea-sickness, but does not speak of its prevention. From twenty-five years' personal experience, and from that of scores of friends and patients, I think I may say that I have discovered a very simple means of preventing sickness, in most cases, in short voyages. This preventive remedy is merely the ingestion of strong coffee some time before embarking.

I discovered the influence of strong coffee quite accidentally. A quarter of a century ago I was traveling on urgent business from Paris to London, and on arriving at Folkestone found that I had an hour or more to dispose of. I went into a *café*, and ordered a cup of *café noir*, the strong black coffee frequently taken on the Continent, with sugar only; this cup I repeated. The weather was awful, the sea tempestuous; nothing but dire necessity would have induced me to start, and I expected to be ill in five minutes, being a wretched sailor. To my utter astonishment, I remained perfectly well throughout a tedious and very rough passage. Indeed, I think I was the only passenger on board who was not ill. This circumstance made me think that perhaps it was the strong coffee that was the cause of the immunity, and subsequent experience has proved that such was the case. To succeed, however, certain precautions are necessary. The coffee should be taken long enough before embarking to ensure its absorption—say one hour, if alone with sugar; about two hours and half, if with milk. Moreover, there should be nothing whatever in the stomach—nothing for the stomach to do. To prevent exhaustion, however, a good, easily-digested meal should be taken about four hours before embarking. The coffee, also, should be good and strong, unmixed with chicory; otherwise it produces no effect. If sickness ensue and food be thrown up, the conditions necessary, according to my experience, to secure immunity, have not been secured, and the non-success is not a real failure. I consider that an infusion of about an ounce and a half of pure coffee-powder is about the quantity necessary. It should be infused (medicinally) in about four ounces of boiling water for ten minutes, say in a warmed mug or jug, poured off and drunk with sugar as *café noir* an hour before leaving, or as *café au lait* (with milk) two hours and a half before. It is best made at home, and put in a bottle, to avoid chicory or English wish-wash.

Contrary to the generally received opinion, I do not think that the stomach being full on going on board is a prevention against sea-sickness. In my experience I find that it promotes and causes it. The poor stomach, full of food, buffetted about, refuses to do its work, and in desperation rejects its contents. I have been at sea nearly a hundred times since then ; thanks to the coffee, I have scarcely ever been sick on short journeys (under ten or twelve hours), and very many persons to whom I have recommended the plan have enjoyed similar immunity. The influence must be that of a brain and nerve tonic, which strengthens the sympathetic nervous system, and renders it less impressionable to liquid shocks. I have published elsewhere the opinion that sea-sickness is probably due, in a measure, to disturbance of the circulation by the irregular motion, and to its influence on the sympathetic nerves. All the blood in the body is irregularly banged about by the motion of the vessel in a rough sea. Animals at sea for the first time are as ill as ourselves, especially dogs, and it cannot be fear, for on land fear does not produce sickness with them or with us, unless very exceptionally.

On longer jonrneys, I advise travelers still to take the coffee in the same way, and then to lie down in their berths. The influence of a strong dose of coffee on the nervous system lasts for eight or ten hours. During this time the body may become accustomed to the motion of the ship. They had better, I should say, take no food, either liquid or solid, until the feeling of thirst or of hunger show itself. For the first (thirst), a mineral water, soda or Apollinaris, with or without brandy or champagne, may be sipped. For the second (hunger), *café au lait*, with or without a little bread, may be taken ; annd that agreeing, curry, as advised by Dr. Kendall, could be tried.

In the continued sickness of long voyages, many patients of mine have derived immense, and often permanent, relief from the injection into the rectum at night of fifteen or twenty drops of laudanum, in an ounce and a half of warm water, to be retained, by means of a small elastic baby injection-ball. If not retained, it can be repeated in half an hour or an hour. Sleep is generally obtained, even in extreme cases; and during sleep the body may become accustomed to the motion, as will coffee. Some patients have told me, or written to me, after long voyages, that this mode of employing laudanum has saved their lives. I have proposed, as have others, the hypodermic injection of morphia; but I have no personal experience of its use, and cannot say whether or not it acts better than the injection of laudanum *per anum.* When on board ship in short passages, I am generally too fearful of personal sickness to be of much use to others, feeling it prudent to remain perfectly quiescent; and I have not made any very long sea voyages.

Probably strong tea, brandy, wine, indeed any powerful nerve-stimulant or sedative, would have the same effect as coffee if taken in the same manner—that is, sufficiently long before going on board to be absorbed, and to leave the stomach empty and quiescent.

Clearly, the usual non-success of remedial agents taken for sea-sickness is owing to the stomach itself refusing to absorb what is put into it, once sickness has commenced. A glass of pure water, instead of being absorbed in a few minutes, will often be thrown up an hour or more afterwards, just as it was taken. I have often known chloroform thrown up half an hour or more after its ingestion, perfuming the ship with its odor. The rectum is not sick, and has not lost its power of absorption.

A naval surgeon, who had passed a life at sea, once told me that what he had found of most benefit in confirmed desperate sickness, was drinking constantly warm or lukewarm water. A jug of warm water and a glass are placed beside the patient, who is told to swallow half a tumblerful when the sickness comes on. It is immediately thrown up, but easily, and by-and-by calm comes. The real remedy, however, is to stay on land, or to return there, if possible; but the coffee plan, judiciously carried out, has shorn the Channel of its horrors in my case, and in that of many others.—*Brit. Med. Jour.*

